# Preoperative left atrial volume index may be associated with postoperative atrial fibrillation in non-cardiac surgery

**DOI:** 10.3389/fcvm.2022.1008718

**Published:** 2022-11-03

**Authors:** Ah Ran Oh, Sung Ho Lee, Jungchan Park, Jong-Hwan Lee, Dahye Cha, Kwangmo Yang, Jin-Ho Choi, Joonghyun Ahn, Ji Dong Sung, Bogeum Choi, Seung-Hwa Lee

**Affiliations:** ^1^Department of Anesthesiology and Pain Medicine, Samsung Medical Center, School of Medicine, Sungkyunkwan University, Seoul, South Korea; ^2^Department of Anesthesiology and Pain Medicine, Kangwon National University Hospital, Chuncheon, South Korea; ^3^Division of Cardiology, Department of Internal Medicine, Kangbuk Samsung Hospital, School of Medicine, Sungkyunkwan University, Seoul, South Korea; ^4^Department of Biomedical Sciences, Ajou University Graduate School of Medicine, Suwon, South Korea; ^5^Center for Health Promotion, Samsung Medical Center, School of Medicine, Sungkyunkwan University, Seoul, South Korea; ^6^Department of Emergency Medicine, Samsung Medical Center, School of Medicine, Sungkyunkwan University, Seoul, South Korea; ^7^Statistics and Data Center, Research Institute for Future Medicine, Samsung Medical Center, Seoul, South Korea; ^8^Rehabilitation and Prevention Center, Heart Vascular Stroke Institute, Samsung Medical Center, School of Medicine, Sungkyunkwan University, Seoul, South Korea; ^9^College of Medicine, Kyung Hee University, Seoul, South Korea; ^10^Department of Biomedical Engineering, Seoul National University College of Medicine, Seoul, South Korea

**Keywords:** non-cardiac surgery, atrial fibrillation, left atrial volume index, echocardiography, postoperative cardiac complications

## Abstract

**Background:**

Postoperative atrial fibrillation (POAF) is related to mortality after non-cardiac surgery. Left atrial volume index (LAVI) is known to be associated with prognosis and development of atrial fibrillation, but it has not been fully investigated in patients undergoing non-cardiac surgery.

**Materials and methods:**

A total of 203,787 consecutive adult patients underwent non-cardiac surgery at our institution between January 2011 and June 2019. After identifying those with available LAVI estimated during preoperative echocardiography, we divided them into those with LAVI higher and lower than 34 mL/m^2^. The primary outcome was incidence of POAF.

**Results:**

A total of 83,097 patients were enrolled in this study. The study patients were divided into the low (57,838 [69.6%]) and high (25,259 [30.4%]) LAVI groups. After an adjustment, higher LAVI was associated with increased incidence of POAF (5.1% vs. 8.1%; odds ratio [OR], 1.33; 95% confidence interval [CI], 1.25–1.41; *p* < 0.001). In 24,549 pairs of propensity-score-matched population, the result was similar (6.2% vs. 7.9%; OR, 1.30; 95% CI, 1.21–1.39; *p* < 0.001). The estimated threshold of LAVI associated with POAF was 36.4 mL/m^2^ with an area under the curve of 0.571. Subgroup analysis in non-thoracic and thoracic surgery showed that the association between preoperative LAVI and POAF significantly interacted with diastolic dysfunction (*p* for interaction < 0.001), and the observed association was valid in patients without diastolic dysfunction.

**Conclusion:**

Preoperative LAVI was shown to be associated with POAF in non-cardiac surgery. Our result needs verification in further studies.

## Introduction

As average age and risk of surgery increase, postoperative complications have become a major health issue (1). Cardiac complications are closely associated with perioperative mortality (2), and new-onset atrial fibrillation following surgery, commonly referred to as postoperative atrial fibrillation (POAF), has been reported to frequently occur even in non-cardiac surgeries (3). POAF in non-cardiac surgery is now widely accepted to potentially lead to long-term consequences such as increased morbidity, mortality, hospital length of stay, and long-term risk of stroke (4, 5).

Left atrial volume index (LAVI) on echocardiography has been shown to be associated with atrial fibrillation in various clinical situations. In addition, LAVI was shown a predictive marker for the occurrence of atrial fibrillation and recurrence after treatment (6–8). In preoperative echocardiographic evaluation of cardiac surgery, LAVI was associated with POAF in various procedures (9, 10). However, this association remains unclear for non-cardiac surgery and may be related to a wide variety of non-cardiac surgeries or clinical events during surgical procedures because the development of POAF can largely vary depending on these factors. In the present study, consecutive adult patients who underwent non-cardiac surgery in a tertiary center between January 2011 and June 2019 were enrolled and divided into two groups based on preoperative LAVI and the incidence of POAF was compared. In addition, whether the significance of this association differed based on subgroup was evaluated.

## Materials and methods

The approval and requirement for written informed consent for this study were waived by the Institutional Review Board of Samsung Medical Center (SMC 2021-06-078) because the registry for this study was curated in de-identified form. The present study was conducted according to the Declaration of Helsinki and reported following the Strengthening the Reporting of Observational Studies in Epidemiology guidelines.

### Data curation, study population, and definitions

The Samsung Medical Center-Non-Cardiac operation, KCT 0006363 (SMC-NoCop) registry was used to identify subjects. SMC-NoCop is a large, single-center, de-identified cohort which consists of 203,787 consecutive adult patients who underwent non-cardiac surgery at Samsung Medical Center, Seoul, South Korea between January 2011 and June 2019. The Clinical Data Warehouse Darwin-C was used to extract data from our institutional electronic archive system which allows investigators to search and retrieve de-identified medical information from electronic hospital records of more than 4 million patients with more than 900 million laboratory findings and 200 million prescriptions. In this system, the mortality outside our institution is verified by a unique personal identification number and consistently updated according to the National Population Registry of the Korea National Statistical Office. From the entire population, we excluded the patients with preoperative atrial fibrillation or those without LAVI estimated from preoperative echocardiography. The patients were divided into high LAVI (≥34 mL/m^2^) and low LAVI (<34 mL/m^2^) groups according to current updated cut-off value (11).

Preoperative variables such as demographic data, preoperative medication, underlying disease, and blood laboratory tests were recorded based on preoperative evaluation by independent investigators. The International Classification of Diseases-10 codes were also used to estimate preoperative Charlson comorbidity index (12). The risk of surgical procedure was stratified according to the European Society of Cardiology/European Society of Anesthesiology guidelines on non-cardiac surgery (13). The diagnosis of heart failure was made by our cardiologists using Framingham criteria and other relevant clinical information (14). These included a history of dyspnea and symptomatic exercise intolerance with signs of pulmonary congestion or peripheral edema, the presence of moist rales on auscultation, or left ventricular enlargement or dysfunction by chest X-ray or echocardiography (15). The patients with heart failure were categorized into heart failure with preserved ejection fraction (HFpEF; ejection fraction > 40%) and heart failure with reduced ejection fraction (HFrEF; ejection fraction ≤ 40%) groups (16).

In our institution, preoperative transthoracic echocardiography including Doppler imaging was selectively obtained for non-cardiac surgery patients. An echocardiographic evaluation is recommended for moderate- to high-risk surgery or for patients with at least one major cardiovascular risk factor such as history of ischemic heart disease, heart failure, stroke, including transient ischemic attack, diabetes mellitus patients on insulin therapy, or chronic kidney disease, based on current guidelines (13). In patients with minor risk factors, transthoracic echocardiography was obtained at the discretion of the attending clinician based on older age or recent presentation of cardiovascular symptoms. Echocardiographic parameters were calculated according to current guidelines (17, 18). Left atrial volume was assessed by the biplane area-length method from apical 4- and 2-chamber views (19). Measurements were obtained in end systole from the frame preceding mitral valve opening, and the volume was indexed for body surface area. The normal value of indexed left atrial volume has been reported to be 20 ± 6 mL/m^2^ (20). The left atrium is directly exposed to left ventricle pressure during diastole, so left atrium size reflects the duration and severity of diastolic dysfunction (21).

### Study endpoints

The primary endpoint was the occurrence of POAF during hospitalization. We followed-up the patients during this period, considering that the risk for POAF peaks at around 48 h after surgery (22). We used postoperative diagnosis based on the International Classification of Diseases-10 codes and reviewed electrocardiogram reading, in-hospital progress notes, nursing charts, discharge notes, and replies for cardiologist consultation. The secondary outcome was POAF that required interventional treatment such as intravenous administration of antiarrhythmic agents such as propafenone, flecainide, amiodarone, diltiazem, or verapamil or patients who required electrical cardioversion for rhythm or rate control.

### Statistical analysis

Baseline characteristics of study patients are presented by number and percentage for categorical variables and the mean ± standard deviation (SD) or median with interquartile range (IQR) for continuous variables. Difference between groups was compared using the chi-square, Fisher’s exact test, *t*-test, or the Mann–Whitney test, as applicable. The incidence of POAF was compared using the logistic regression analysis and reported with odds ratio (OR) and 95% confidence interval (CI). The multivariable adjustment included age, hypertension, diabetes, chronic kidney disease, coronary aery disease, heart failure, general anesthesia, and preoperative use of beta blocker and calcium channel blocker. In addition, a propensity score matching to generate 1:1 individually matched populations without replacement was performed. Patients were matched on all variables, and propensity score using a nearest-neighbor matching technique. The propensity score was calculated using the logistic regression model with patients with high LAVI as the dependent variable. A greedy matching technique was employed to match patients using the logit of the propensity-score matching with a caliper width of 0.1 difference of the logit of the propensity score. After propensity score matching, 24,549 pairs were obtained with an absolute standard mean deviation (ASD) of <10% which was deemed a successful balance between the groups. Based on the sample size, the power of the analysis was 0.79 when OR was 1.1, and 0.99 when OR was >1.2 (23). In addition, the effects of unmeasured confounding factors were calculated. In this method, we evaluated the significance of the observed association between LAVI and POAF assuming the prevalence of unmeasured confounding factor was 40% (24). Subgroup analysis was performed in non-thoracic and thoracic surgery to evaluate whether the observed association interacted with relevant variables such as age, sex, hypertension, diabetes, coronary artery disease, chronic kidney disease, general anesthesia, emergency operation, intraoperative blood transfusion, intraoperative infusion of inotropic drugs, and diastolic dysfunction. The receiver operating characteristic (ROC) curve was constructed to estimate the threshold of LAVI associated with POAF and compute its specificity and sensitivity. The difference between two areas under the ROC curves was tested with the z test. All analyses were performed using R 4.1.1^1^ (Vienna, Austria).

## Results

### Baseline characteristics

A total of 203,787 patients are in the SMC-NoCop registry, and 1,923 (0.9%) patients with preoperatively detected atrial fibrillation and 118,767 (58.3%) patients without estimated LAVI during preoperative evaluation were excluded from the present study. The remaining 83,097 patients were enrolled in the study and divided into two groups based on LAVI: low LAVI [<34 mL/min^2^, *n* = 57,838 (69.6%)] and high LAVI [≥ 34 mL/min^2^, *n* = 25,259 (30.4%)] groups (11). The baseline characteristics of the two groups are summarized in Table 1. Subjects in the high LAVI group were more frequently male, older, and had higher incidence of comorbidities. Other parameters on preoperative echocardiography are summarized in Table 2.

**TABLE 1:** Preoperative variables according to left atrial volume index (LAVI).

	Entire population				Propensity-score-matched population		
	Low LAVI (*N* = 57,838)	High LAVI (*N* = 25,259)	*P*-value	ASD	Low LAVI (*N* = 24,549)	High LAVI (*N* = 24,549)	ASD
Male	27522 (47.6)	11819 (46.8)	0.04	1.6	11507 (46.9)	11514 (46.9)	0.1
Age	60.4 (±12.0)	65.1 (±10.9)	<0.001	41	65.2 (±10.4)	64.8 (±10.8)	3.1
Hypertension	19905 (34.4)	12424 (49.2)	<0.001	30.3	11815 (48.1)	11835 (48.2)	0.2
Diabetes	7207 (15.9)	5232 (20.7)	<0.001	12.4	4837 (19.7)	4921 (20.0)	0.9
Current alcohol	8899 (15.4)	3592 (14.2)	<0.001	3.3	3489 (14.2)	3540 (14.4)	0.6
Current smoking	3270 (5.7)	1362 (5.4)	0.14	1.1	1255 (5.1)	1322 (5.4)	1.2
Chronic kidney disease	898 (1.6)	1255 (5.0)	<0.001	19.3	809 (3.3)	905 (3.7)	2.1
**Preoperative medication**							
Beta blocker	3337 (5.8)	3144 (12.4)	<0.001	23.4	2625 (10.7)	2709 (11.0)	1.1
Calcium channel blocker	8209 (14.2)	6231 (24.7)	<0.001	26.7	5650 (23.0)	5682 (23.1)	0.3
**Previous disease**							
Stroke	1518 (2.6)	990 (3.9)	<0.001	7.3	909 (3.7)	923 (3.8)	0.3
Coronary artery disease	1863 (3.2)	1395 (5.5)	<0.001	11.3	1227 (5.0)	1251 (5.1)	0.4
Myocardial infarction	358 (0.6)	374 (1.5)	<0.001	8.5	218 (0.9)	323 (1.3)	4.1
**Coronary revascularization**							
Percutaneous intervention	1380 (2.4)	1093 (4.3)	<0.001	10.8	843 (3.4)	984 (4.0)	3.0
Bypass graft	135 (0.2)	206 (0.8)	<0.001	8.1	97 (0.4)	173 (0.7)	4.2
Heart failure	114 (0.2)	276 (1.1)	<0.001	11.2	101 (0.4)	135 (0.5)	2.0
Arrhythmia	389 (0.7)	342 (1.4)	<0.001	6.8	271 (1.1)	279 (1.1)	0.3
Peripheral artery disease	212 (0.4)	179 (0.7)	<0.001	4.7	136 (0.6)	152 (0.6)	0.9
Aortic disease	279 (0.5)	241 (1.0)	<0.001	5.6	191 (0.8)	21 (0.9)	0.9
Valvular heart disease	54 (0.1)	112 (0.4)	<0.001	6.8	50 (0.2)	71 (0.3)	1.7
Chronic obstructive pulmonary disease	1756 (3.0)	753 (3.0)	0.69	0.3	705 (2.9)	734 (3.0)	0.7
**Operative variables**							
General anesthesia	51422 (88.9)	20901 (82.7)	<0.001	17.7	20583 (83.8)	20523 (83.6)	0.7
Emergency operation	1720 (3.0)	962 (3.8)	<0.001	4.6	881 (3.6)	891 (3.6)	0.2
Operation duration, min	147.2 (±105.4)	148.5 (±111.2)	0.11	1.2	148.3 (±107.9)	149.3 (±111.4)	1.0
Surgical risk			<0.001	4.8			1.0
Mild	20912 (36.2)	8737 (34.6)			8547 (34.8)	8534 (34.8)	
Intermediate	32074 (55.5)	14101 (55.8)			13708 (55.8)	13648 (55.6)	
High	4852 (8.4)	2421 (9.6)			2294 (9.3)	2367 (9.6)	
Inotropic drug infusion	6616 (11.4)	3422 (13.5)	<0.001	6.4	3158 (12.9)	3297 (13.4)	1.7
Blood transfusion	1945 (3.4)	1333 (5.3)	<0.001	9.4	1036 (4.2)	1270 (5.2)	4.5
Red blood cell	1890 (3.3)	1268 (5.0)	<0.001	8.8	1017 (4.1)	1206 (4.9)	3.7
Cryoprecipitate	137 (0.2)	149 (0.6)	<0.001	5.5	68 (0.3)	146 (0.6)	4.8
Fresh frozen plasma	412 (0.7)	341 (1.4)	<0.001	6.3	206 (0.8)	329 (1.3)	4.8
Platelet concentrate	66 (0.1)	66 (0.3)	<0.001	3.4	36 (0.1)	61 (0.2)	2.3
**Surgery types**							
Neuroendocrine	1598 (2.8)	726 (2.9)	0.39	0.7	687 (2.8)	714 (2.9)	0.7
Lung	8048 (13.9)	2700 (10.7)	<0.001	9.8	3056 (12.4)	2669 (10.9)	4.9
Head and neck	4304 (7.4)	2177 (8.6)	<0.001	4.3	1971 (8.0)	2131 (8.7)	2.4
Breast	9208 (15.9)	2166 (8.6)	<0.001	22.5	2429 (9.9)	2156 (8.8)	3.8
Stomach	5891 (10.2)	2130 (8.4)	<0.001	6	2089 (8.5)	2110 (8.6)	0.3
Hepatobiliary	4326 (7.5)	2368 (9.4)	<0.001	6.8	2086 (8.5)	2320 (9.5)	3.3
Colorectal	5758 (10.0)	2478 (9.8)	0.53	0.5	2347 (9.6)	2441 (9.9)	1.3
Urology	5257 (9.1)	2709 (10.7)	<0.001	5.5	2680 (10.9)	2641 (10.8)	0.5
Gynecology	1179 (2.0)	584 (2.3)	0.001	1.9	564 (2.3)	574 (2.3)	0.3
Bone and skin etc.	12269 (21.2)	7221 (28.6)	<0.001	17.1	6640 (27.0)	6793 (27.7)	1.4

Data are presented as n (%) or mean (±standard deviation). Surgical risk was stratified according to 2014 European Society of Cardiology/European Society of Anesthesiology guidelines.

**TABLE 2:** Echocardiographic parameters.

	Entire population			
	Low LAVI (*N* = 57,838)	High LAVI (*N* = 25,259)	*P*-value	ASD
Ejection fraction	63.9 (6.1)	64.6 (6.5)	<0.001	10.7
E/e’ ratio	8.5 (14.7)	10.3 (4.0)	<0.001	16.6
E/A ratio	0.91 (0.58)	0.90 (0.47)	0.02	1.8
Deceleration time, ms	240.3 (54.8)	243.4 (58.0)	<0.001	5.4

### Postoperative atrial fibrillation

The overall incidence of POAF was 6.0% (4,998/83,097) and 4.9% (4,087/83,097) needed interventional treatment. Subjects in the high LAVI group showed increased incidence of POAF (5.1 vs. 8.1%; OR, 1.63; 95% CI, 1.54–1.73; *p* < 0.001; Table 3). After adjustment, the high LAVI group consistently showed an increased incidence of POAF (OR, 1.33; 95% CI, 1.25–1.41; *p* < 0.001). The risk of POAF that required interventional treatment was also increased in the high LAVI group (4.1 vs. 6.7%; OR, 1.32; 95% CI, 1.23–1.41; *p* < 0.001; Table 3).

**TABLE 3:** Incidence of postoperative atrial fibrillation.

	Low LAVI	High LAVI	Unadjusted OR (95% CI)	*P*-value	Adjusted OR (95% CI)	*P*-value
**Entire population**	*N* = 57,838	*N* = 25,259				
Atrial fibrillation	2955 (5.1)	2043 (8.1)	1.63 (1.54–1.73)	<0.001	1.33 (1.25–1.41)	<0.001
Treatment required	2389 (4.1)	1698 (6.7)	1.67 (1.57–1.78)	<0.001	1.32 (1.23–1.41)	<0.001
**Propensity-score-matched population**	*N* = 24,549	*N* = 24,549				
Atrial fibrillation	1518 (6.2)	1934 (7.9)			1.30 (1.21–1.39)	<0.001
Treatment required	1274 (5.2)	1604 (6.5)			1.28 (1.18–1.38)	<0.001

Data are presented as n (%). Multivariable adjustment included age, hypertension, diabetes, chronic kidney disease, coronary artery disease, heart failure, general anesthesia, and preoperative use of beta blocker and calcium channel blocker.

After propensity score matching, 24,549 pairs of patients with well-balanced variables were generated. The high LAVI group was consistently associated with increased incidence of POAF (6.2 vs. 7.9%; OR, 1.30; 95% CI, 1.21–1.39; *p* < 0.001 for overall POAF and 5.2 vs. 6.5%; OR, 1.28; 95% CI, 1.18–1.38; *p* < 0.001 for treatment required; Table 3). The observed association between LAVI and POAF remained significant under any circumstance regardless of unmeasured confounding factors (Supplementary Table 1).

**FIGURE 1:**
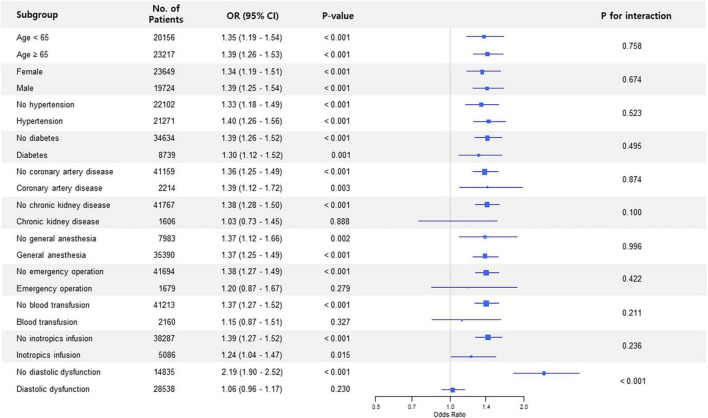
Forest plot of subgroup analysis in non-thoracic surgery.

**FIGURE 2:**
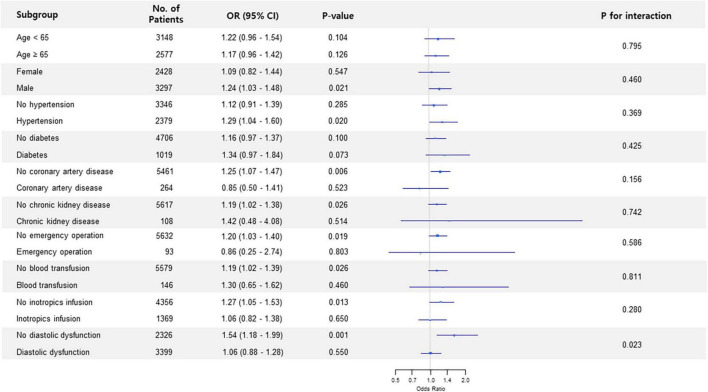
Forest plot of subgroup analysis in thoracic surgery.

Subgroup analysis in non-thoracic surgery showed that the association between preoperative LAVI and POAF significantly interacted with diastolic dysfunction (*p* for interaction < 0.001) (Figure 1). The observed association was valid in patients without diastolic dysfunction but was non-significant in subjects who had diastolic dysfunction. This trend in diastolic dysfunction was also retained in subgroup analysis for thoracic surgery (*p* for interaction = 0.023) (Figure 2).

**FIGURE 3:**
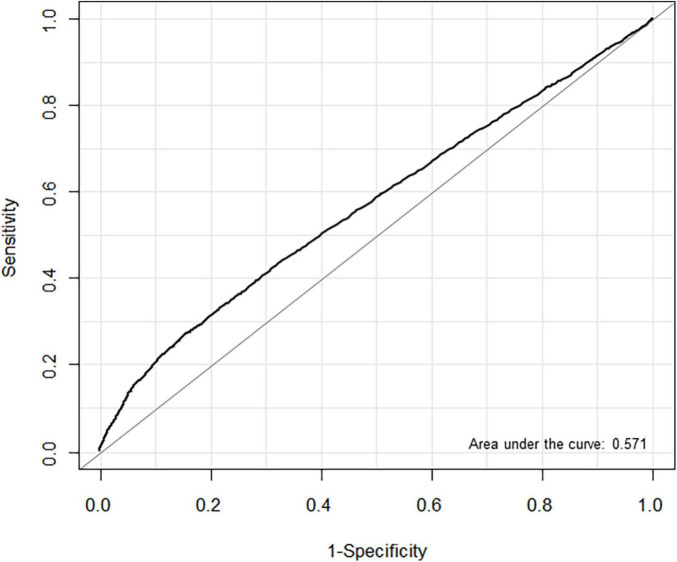
Receiver operating characteristic (ROC) curves for left atrial volume index (LAVI) associated with postoperative atrial fibrillation (POAF) in non-cardiac surgery.

In the ROC analysis, the estimated threshold of LAVI associated with POAF in entire cohort was 36.4 mL/min^2^ with an area under the curve (AUC) of 0.571. Using this threshold, the sensitivity and specificity were 33.9 and 77.6%, respectively (Figure 3). We also evaluated the predictive performance of LAVI in different subgroups. In patients with heart failure, the ROC analysis showed that AUC value was 0.611 for HFpEF (cut-off 46.3 mL/min^2^, sensitivity 59.3%, specificity 63.0%) and 0.606 for HFrEF (cut-off 36.9 mL/min^2^, sensitivity 100%, specificity 37.8%) (Supplementary Figure 1). The AUC value in non-thoracic and thoracic surgery was 0.590 (cut-off 37.3 mL/min^2^, sensitivity 34.7%, specificity 79.4%) and 0.543 (cut-off 36.6 mL/min^2^, sensitivity 24.2%, specificity 83.4%), respectively (Supplementary Figure 2). The AUC calculated for LAVI in non-thoracic surgery was significantly higher than that in thoracic surgery (*P* < 0.001).

## Discussion

In the present study, high preoperative LAVI showed association with POAF in patients undergoing non-cardiac surgery. The overall incidence of POAF was 6.0% and 4.9% needed interventional treatment. Subgroup analysis based on perioperative variables showed this association was valid only in patients without diastolic dysfunction in non-thoracic and thoracic surgery.

Left atrial volume index is a reliable and readily measurable indicator for the burden of cardiovascular disease. In cardiac surgery, LAVI was shown the strongest predictor of POAF (25). Although an exact mechanism how left atrial enlargement affects development of atrial fibrillation remains unclear, a possible explanation may be associated with atrial stretch which activates atrial fibrosis and increases the atrial effective refractory period leading to atrial arrhythmia (26, 27). In non-cardiac surgery, the association between LAVI and POAF has been evaluated in several studies; however, those studies were limited by surgery type and showed conflicting results (28, 29). Furthermore, most of the previous studies predate recently accepted upper normal value of LAVI (34 mL/m^2^) and analysis performed based on a significantly lower value (11). In the present study, LAVI was assessed with a revised reference value and preoperatively increased LAVI was associated with POAF in a broad non-cardiac surgical population.

Preexisting conditions such as older age, male sex, and history of cardiovascular disease have been previously reported association with POAF (30, 31). However, these are rather general risk factors for cardiac complications and are difficult to apply in a clinical practice to predict POAF. Clinically, LAVI is a value that can be measured objectively and reflects pathophysiologic change directly associated with atrial fibrillation. Furthermore, in the present study, LAVI showed association with the development of POAF that requires an intervention. POAF that requires urgent treatment is a commonly found clinical situation for patients with significant heart disease. Clinically, the results of this study indicate the possibility that LAVI can be used as a relatively more specific marker for POAF prediction compared with other general cardiac risk factors.

The estimated LAVI threshold associated with POAF was 36.4 mL/min^2^, close to the revised reference limit of LAVI (11). However, the overall predictive accuracy was low (AUC = 0.571) compared with the results from cardiac surgeries (32, 33). Also, the predictive performance of LAVI in HFpEF (AUC = 0.611) and HFrEF (AUC = 0.606) were similar, although HFpEF and HFrEF represent two distinct disease entities in the heart failure spectrum (34, 35). These results are possibly because development of POAF involves multifactorial mechanisms in non-cardiac surgeries and indicate the limitation of predicting POAF solely by preoperative echocardiographic findings.

To evaluate the usefulness of single value of LAVI in non-cardiac surgery, we divided the surgery type into non-thoracic and thoracic surgery and investigated the cut-off value of LAVI associated with POAF. The non-thoracic group showed a larger cut-off value with significantly higher AUC than the thoracic group. This discrepancy may be attributed to the fact that the two types of surgeries have different incidence and risk factors for POAF (36, 37). Therefore, LAVI may have a different threshold and predictive accuracy in each subgroup. Nevertheless, the optional cut-off value of LAVI for predicting POAF in non-cardiac surgery has yet to be evaluated well, so further multicenter and larger cohort studies are required to verify our results.

In addition, subgroup analysis was conducted in non-thoracic and thoracic surgery based on perioperative factors. The association between LAVI and POAF was significantly affected by diastolic dysfunction, and the association was not observed in patients with diastolic dysfunction in both types of surgeries. Diastolic dysfunction is associated with an increasing stretch in pulmonary veins due to increased left atrial pressure (38), which elevates the arrhythmogenic activity of the pulmonary veins (39). These effects may have outweighed the effect of preoperative left atrial volume, so the observed association might have been significant only in patients without diastolic dysfunction. The subgroup analysis results need further verification in prospectively collected data.

Left atrial volume index is not the only echocardiographic parameter associated with diastolic dysfunction and higher incidence of POAF (40). Therefore, other parameters were compared between the two groups, and the indicators of diastolic dysfunction such as E/e’ ratio and deceleration were significantly higher in the high LAVI group. In previous studies, LAVI was also reportedly not an independent predictor of POAF in patients with well-preserved left ventricular ejection fraction (29–41). In the present study, the association between LAVI and POAF was significant although the ejection fraction tended to be preserved in both groups. Therefore, other echocardiographic parameters also need further investigation regarding the development of POAF in non-cardiac surgery.

The present study had several limitations that should be considered when interpreting the results. First, because this was a retrospective study, unmeasured confounding factors may have affected the results despite rigorous statistical adjustment using propensity score matching. Despite our effort to exclude patients with preoperative atrial fibrillation, the patients with paroxysmal atrial fibrillation not specified in medical records may have been included in this study. Second, only patients who had echocardiography before surgery were included. Therefore, the study cohort may have had more cardiovascular disease which may have caused selection bias and overestimation of the incidence of POAF in non-cardiac surgery. Third, echocardiography was not performed after the surgery; thus, there is no information regarding postoperative LAVI, which may have contributed to the development of POAF. Fourth, we only observed POAF that occurred during a short period of hospitalization after surgery. However, surgical stress can persist even after patients are discharged and induce POAF for a long-term period. This short-term follow-up may have underestimated the risk of POAF. Despite these limitations, an association was found between preoperative LAVI and POAF using large real-world data. Preoperative echocardiographic measures could be used to identify patients who are at a high risk for developing POAF and may provide useful prognostic information for individual patients. Also, for these high-risk populations, proven perioperative interventions aimed at reducing POAF might be very cost-effective in non-cardiac surgery.

## Conclusion

The present study results showed the increased preoperative LAVI on echocardiography was associated with the development of POAF in non-cardiac surgery. Preoperative LAVI may be helpful in predicting POAF; however, further verification is needed.

## Data availability statement

The data we used for this study was curated using Clinical Data Warehouse (CDW) which psuedonomynize the data from our institutional electronic medical records. So, our data is de-identified by eliminating all identifiable variables such as name, social security number, hospital number, and etc. However, it is illegal to open this data to the public without restriction. Regarding the availability of our data, please contact jong-hwan.park@samsung.com, the head of our institutional data security department.

## Author contributions

JP and SL: study conception and design. DC, KY, JA, and BC: data acquisition and analysis. AO and SL: writing of the manuscript. J-HL, J-HC, and JS: study supervision and data interpretation. All authors approved the manuscript.

## Footnotes

1
http://www.R-project.org/

## Supplementary material

Supplementary Figure 1 Receiver operating characteristic (ROC) curves for left atrial volume index (LAVI) associated with postoperative atrial fibrillation (POAF) in **(A)** HFpEF and **(B)** HFrEF subgroups.

Supplementary Figure 2 Receiver operating characteristic (ROC) curves for left atrial volume index (LAVI) associated with postoperative atrial fibrillation (POAF) in **(A)** non-thoracic and **(B)** thoracic surgery.
