# Supplementary material for: Preoperative left atrial volume index may be associated with postoperative atrial fibrillation in non-cardiac surgery
Source: Front Cardiovasc Med. 2022 Nov 3;9:1008718. doi: 10.3389/fcvm.2022.1008718 (PMC9669716; doi:10.3389/fcvm.2022.1008718)
Supplement: Supplementary file 1 [file Table_1.DOCX]

| **Supplementary Table 1.** Effect of an unmeasured confounder on hazard ratio of perioperative adverse cardiac event for one-year mortality in the propensity-score-matched population. | | | | | | | | |
| --- | --- | --- | --- | --- | --- | --- | --- | --- |
|  |  | **OR*_ZY_*_\|_*_X_*** | | | | | | |
|  |  | **1.5** | **2** | **2.5** | **3** | **3.5** | **4** | |
| OR_zx_ | 0.3 | 1.62 (1.50-1.74) | 1.78 (1.65-1.91) | 1.91 (1.78-2.06) | 2.03 (1.88-2.19) | 2.13 (1.98-2.30) | | 2.22 (2.06-2.39) |
|  | 0.4 | 1.54 (1.44-1.66) | 1.66 (1.54-1.78) | 1.75 (1.63-1.88) | 1.83 (1.70-1.97) | 1.90 (1.77-2.04) | | 1.96 (1.82-2.11) |
|  | 0.5 | 1.50 (1.39-1.61) | 1.58 (1.47-1.70) | 1.65 (1.53-1.77) | 1.70 (1.58-1.83) | 1.75 (1.63-1.88) | | 1.79 (1.67-1.93) |
|  | 0.6 | 1.46 (1.36-1.57) | 1.52 (1.42-1.63) | 1.57 (1.46-1.68) | 1.61 (1.50-1.73) | 1.64 (1.53-1.76) | | 1.67 (1.55-1.79) |
|  | 0.7 | 1.43 (1.34-1.54) | 1.47 (1.37-1.58) | 1.51 (1.40-1.62) | 1.54 (1.43-1.65) | 1.56 (1.45-1.67) | | 1.58 (1.47-1.70) |
| Prevalence of unmeasured confounder = 40% | | |  |  |  |  | |  |
| Numbers represent ORs (including 95% CIs). | |  |  |  |  |  | |  |
| HR, hazard ratio; X: dichotomous exposure measure, y dichotomous outcome measure, z : potential dichotomous confounder. | | | | | | | | |
| ORZX indicates the association (OR) between the unmeasured confounder and left atrial volume index. | | | | | | | | |
| ORZY\|X indicates the association (OR) between the unmeasured confounder and postoperative atrial fibrillation. | | | | | | | | |
