# Supplementary figures and images for: Preoperative left atrial volume index may be associated with postoperative atrial fibrillation in non-cardiac surgery
Source: Front Cardiovasc Med. 2022 Nov 3;9:1008718. doi: 10.3389/fcvm.2022.1008718 (PMC9669716; doi:10.3389/fcvm.2022.1008718)

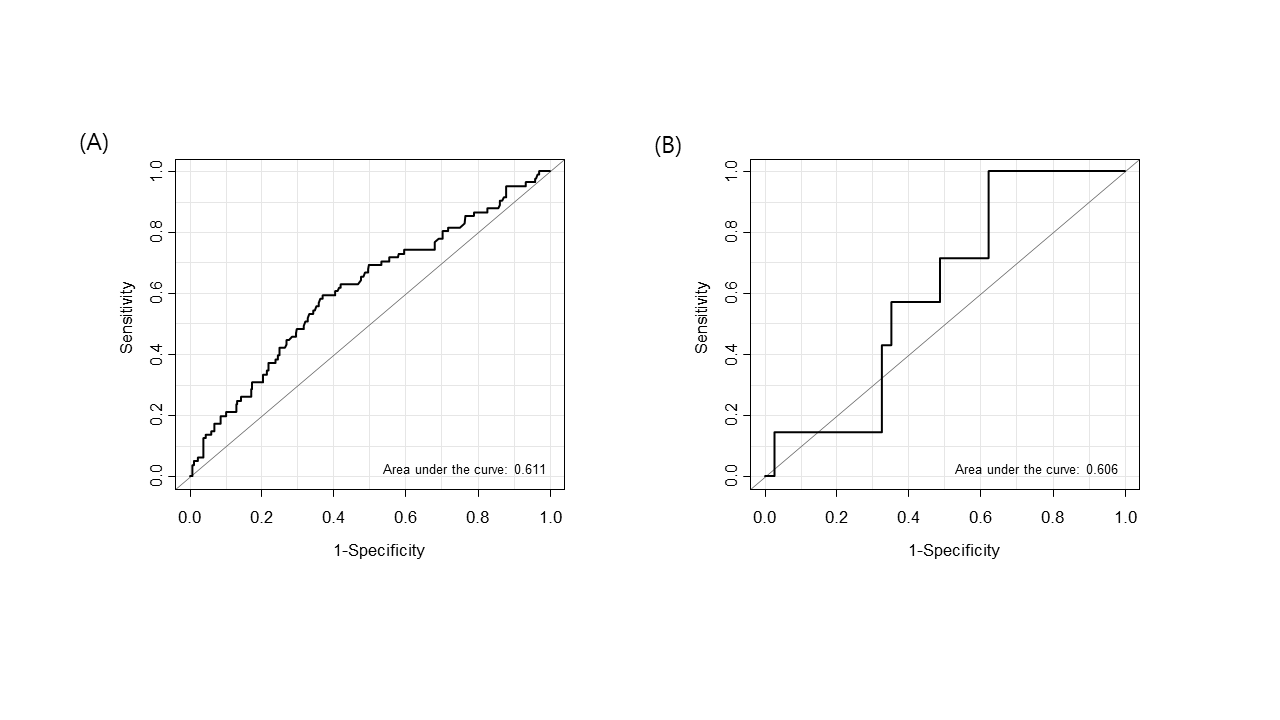

Supplement: Supplementary Figure 1 — Receiver operating characteristic (ROC) curves for left atrial volume index (LAVI) associated with postoperative atrial fibrillation (POAF) in (A) HFpEF and (B) HFrEF subgroups. [file Image_1.TIF]

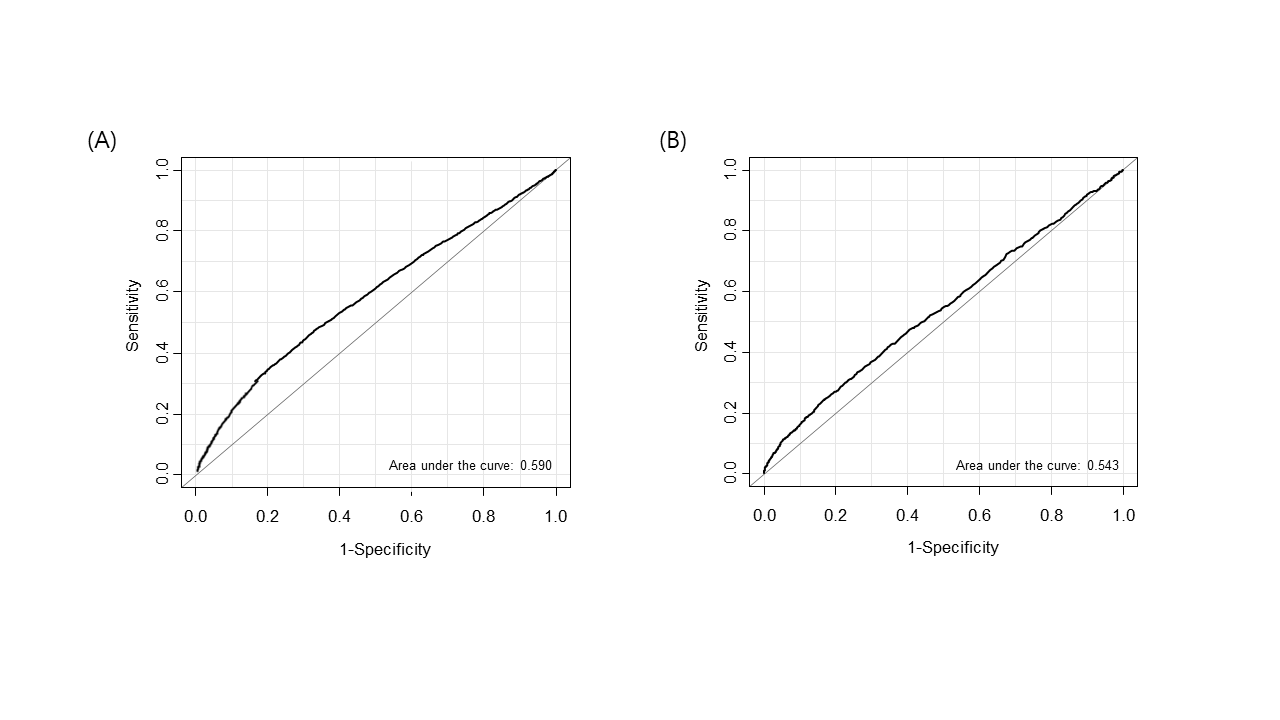

Supplement: Supplementary Figure 2 — Receiver operating characteristic (ROC) curves for left atrial volume index (LAVI) associated with postoperative atrial fibrillation (POAF) in (A) non-thoracic and (B) thoracic surgery. [file Image_2.TIF]
